# Expression of *MdCCD7* in the scion determines the extent of sylleptic branching and the primary shoot growth rate of apple trees

**DOI:** 10.1093/jxb/erx404

**Published:** 2017-11-28

**Authors:** Toshi M Foster, Susan E Ledger, Bart J Janssen, Zhiwei Luo, Revel S M Drummond, Sumathi Tomes, Sakuntala Karunairetnam, Chethi N Waite, Keith A Funnell, Ben M van Hooijdonk, Ali Saei, Alla N Seleznyova, Kimberley C Snowden

**Affiliations:** 1 The New Zealand Institute for Plant & Food Research Limited, Palmerston North, New Zealand; 2 Faculty of Health Sciences, Charles Perkins Centre, The University of Sydney, NSW, Australia; 3 The New Zealand Institute for Plant & Food Research Limited, Auckland, New Zealand; 4 The New Zealand Institute for Plant & Food Research Limited, Havelock North Havelock North, New Zealand; 5 The New Zealand Institute for Plant & Food Research Limited, Kerikeri, New Zealand

**Keywords:** *CAROTENOID CLEAVAGE DIOXYGENASE (CCD)*, *Malus*×*domestica*, shoot growth rate, strigolactone, sylleptic branching

## Abstract

Branching has a major influence on the overall shape and productivity of a plant. Strigolactones (SLs) have been identified as plant hormones that have a key role in suppressing the outgrowth of axillary meristems. *CAROTENOID CLEAVAGE DIOXYGENASE* (*CCD*) genes are integral to the biosynthesis of SLs and are well characterized in annual plants, but their role in woody perennials is relatively unknown. We identified *CCD7* and *CCD8* orthologues from apple and demonstrated that *MdCCD7* and *MdCCD8* are able to complement the Arabidopsis branching mutants *max3* and *max4* respectively, indicating conserved function. RNAi lines of *MdCCD7* show reduced gene expression and increased branching in apple. We performed reciprocal grafting experiments with combinations of *MdCCD7* RNAi and wild-type ‘Royal Gala’ as rootstocks and scion. Unexpectedly, wild-type roots were unable to suppress branching in *MdCCD7* RNAi scions. Another key finding was that *MdCCD7* RNAi scions initiated phytomers at an increased rate relative to the wild type, resulting in a greater node number and primary shoot length. We suggest that localized SL biosynthesis in the shoot, rather than roots, controls axillary bud outgrowth and shoot growth rate in apple.

## Teaser

This is the first case of wild-type roots being unable to suppress branching in a strigolactone-deficient scion. *MdCCD7* RNAi primary shoots also exhibited an increased growth rate relative to the wild type.

## Introduction

Plant architecture is largely dictated by the activities of meristems (Hallé *et al.*, 1978; Steeves and Sussex, 1989). The shoot apical meristem (SAM) forms the primary axis by initiating new phytomers: repeating units of leaf, axillary meristem, node, and internode. The activity of axillary meristems determines the pattern of branching, which contributes greatly to the overall shape of a plant. In annual plants, axillary meristems form and some of these grow out into secondary shoots, a process that is highly plastic and influenced by genetic, developmental, environmental, and nutritional factors, and interactions with surrounding plants (Khayat and Zieslin, 1982; Kim *et al.*, 2010; Huché-Thélier *et al.*, 2011; Pierik and Testerink, 2014; Drummond *et al.*, 2015). In perennial species, branching is even more complex because axillary meristems can have multiple fates: extending into a shoot without a dormant period (sylleptic shoot), developing into a vegetative shoot after a dormant period (proleptic shoot), developing into a floral bud that opens the following spring, or remaining dormant indefinitely (Costes *et al.*, 2014). In addition, environmental signals such as temperature and day length, planting density, internal competition for carbon between buds, and the effects of pruning and plant manipulation all integrate to regulate the outgrowth of axillary meristems and buds (Girault *et al.*, 2008, 2010; Henry *et al.*, 2011; Rabot *et al.*, 2012; Djennane *et al.*, 2014; Fanwoua *et al.*, 2014).

Plant hormones regulate the outgrowth of axillary meristems through a series of complex interactions (reviewed by Domagalska and Leyser, 2011; Müller and Leyser, 2011; Janssen *et al.*, 2014; Rameau *et al.*, 2015). Auxin has long been implicated in the mechanism of apical dominance over axillary meristems (Thimann and Skoog, 1933; Snow, 1937; Sachs and Thimann, 1964). More recently, genetic mutants that show increased branching have led to the discovery of the newest class of plant hormones, strigolactones (SLs) (Gomez-Roldan *et al.*, 2008; Umehara *et al.*, 2008). These mutants include the *more axillary growth* (*max*) mutants of Arabidopsis (*Arabidopsis thaliana*) (Sorefan *et al.*, 2003; Stirnberg *et al.*, 2002; Turnbull *et al.*, 2002), the *ramosus* (*rms*) mutants of pea (*Pisum sativum*) (Beveridge, 2000; Morris *et al.*, 2001; Rameau *et al.*, 2002), the *decreased apical dominance* (*dad*) mutants of petunia (*Petunia hybrida*) (Napoli, 1996; Napoli and Ruehle, 1996), and the *dwarf* (*d*) and *high-tillering dwarf1* (*htd1*) mutants of rice (*Oryza sativa*) (Zou *et al.*, 2006; Arite *et al.*, 2007).

The biosynthesis of SLs is still not completely understood, but includes the isomerization of a carotenoid precursor by DWARF27, followed by cleavage and cyclization to give carlactone by two *CAROTENOID CLEAVAGE DIOXYGENASE* (*CCD*) genes, *CCD7* and *CCD8* (Alder *et al.*, 2012). Later steps of biosynthesis may vary by species and final products made, but include a cytochrome P450 [e.g. MAX1 in Arabidopsis and Os01g077900 and Os01g0701400 in rice (Zhang *et al.*, 2014)], an oxidoreductase-like enzyme [LATERAL BRANCHING OXIDASE (Brewer *et al.*, 2016], and a sulphotransferase [LOW GERMINATION STIMULANT1 (Gobena *et al.*, 2017)].

*CCD7* and *CCD8* genes have been identified and corresponding mutant phenotypes have been well characterized in a number of species. *MAX3*, *RMS5*, *DAD3*, and *D17/HTD1* are orthologous genes encoding CCD7 (Booker *et al.*, 2004; Johnson *et al.*, 2006; Zou *et al.*, 2006; Drummond *et al.*, 2009), and *MAX4*, *RMS1*, *DAD1*, and *D10* are orthologous genes encoding CCD8 (Sorefan *et al.*, 2003; Snowden *et al.*, 2005; Arite *et al.*, 2007). Grafting experiments have demonstrated that a wild-type (WT) rootstock can restore normal branching patterns in *max3*, *max4*, *rms5*, *rms1*, *dad3*, and *dad1* mutants that are deficient in *CCD7* or *CCD8*, indicating that these genes are necessary to synthesize a long-range signal that inhibits branching (Beveridge *et al.*, 1994; Napoli, 1996; Foo *et al.*, 2001; Morris *et al.*, 2001; Turnbull *et al.*, 2002; Sorefan *et al.*, 2003; Simons *et al.*, 2007). This SL signal can originate in roots, but more complex grafting experiments also indicate that sufficient signal to suppress branching can also be stem derived and only moves acropetally (Napoli, 1996; Foo *et al.*, 2001; Turnbull *et al.*, 2002; Simons *et al.*, 2007).

The phenotypes resulting from SL deficiency are well characterized in annual plants, but less so in woody perennials. Grafting experiments involving combinations of SL-deficient scion and roots are highly informative, but have never been performed in any perennial fruit or nut crop. Given that many of these crops are routinely grafted onto clonal rootstocks, it is important to understand the effects of SL deficiency in scion and rootstocks. Knockdown of the kiwifruit (*Actinidia chinensis*) *CCD8* orthologue resulted in an increased number of branches (Ledger *et al.*, 2010). SL pathway genes have been identified in poplar (*Populus*), and knockdown of the poplar *CCD8* orthologues resulted in short, highly branched trees (Czarnecki *et al.*, 2014; Muhr *et al.*, 2016). In willow (*Salix* spp.), an allelic variant of *SxMAX4* that does not fully complement the *max4* mutant was associated with increased branching after coppicing (Salmon *et al.*, 2014). The identification of such allelic variants can be useful in developing new germplasm.

*Apple* (*Malus*×*domestica*) is a deciduous woody perennial fruit tree grown in commercial orchards, home gardens, and in the wild. In commercial orchards, tree growth is managed to provide optimal light interception essential for high yields. Maintaining optimal shoot architecture is time consuming and costly; therefore, knowledge about the genes regulating bud outgrowth are of importance to breeding programmes. To this end, we identified apple orthologues of *CCD7* and *CCD8*, and demonstrated that they are able to complement *max3* and *max4*, respectively, and that RNAi knockdown of *MdCCD7* in apple resulted in increased sylleptic branching. Reciprocal grafting experiments with RNAi *MdCCD7* and WT scions and rootstocks gave an unexpected result: WT roots were unable to rescue normal branching patterns in RNAi *MdCCD7* scions. Our results suggest that localized SL biosynthesis in the shoot, but not in roots, controls sylleptic branching in apple trees.

## Materials and methods

### Identification and cloning of *MdCCD7* and *MdCCD8* genes

**Fig. 1.**
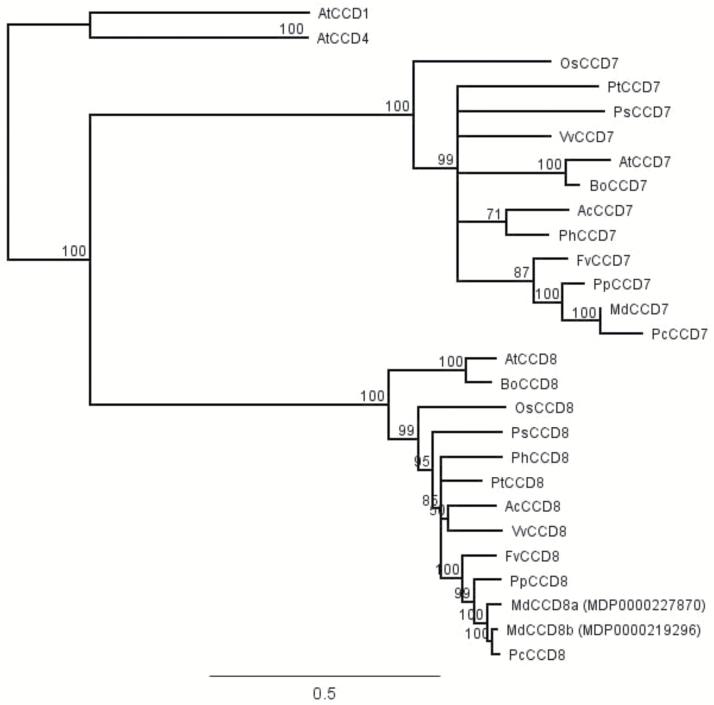
Unrooted phylogenetic tree of the *CAROTENOID CLEAVAGE DIOXYGENASE* (*CCD*) gene family. Only full-length members of the family are included. The predicted sequences were aligned using ClustalW in Geneious (version 10). Phylogenetic relationships were calculated using the maximum-likelihood principle, and bootstrap values with 500 replicates were determined using Geneious. The scale bar is the number of substitutions per site. Accession numbers for the sequences are as follows: *Actinidia chinensis* AcCCD7 (ADP37985.1), AcCCD8 (ADP37984.1); *Arabidopsis thaliana* AtCCD1 (AT3G63520), AtCCD4 (AT4G19170), AtCCD7 (AT2G44990.1), AtCCD8 (AT4G32810); *Brassica oleracea* BoCCD7 (XP_013635602.1), BoCCD8 (XP_013589279.1); *Fragaria vesca* FvCCD7 (XP_004306976.2), FvCCD8 (XP_011458989.1); *Malus×domestica* MdCCD7 (MF034498), MdCCD8a (XP_008378214.1), MdCCD8b (XP_008352014.1); *Oryza sativa* OsCCD7 (LOC_Os04g46470.1), OsCCD8 (XP_015642760.1); *Petunia×hybrida* PhCCD7 (ACY01408.1), PhCCD8 (AAW33596.1); *Pisum sativum* PsCCD7 (ABD67496.2), PsCCD8 (AAS66906.1); *Populus trichocarpa* PtCCD7 (XP_006375244.1), PtCCD8 (XP_002324797.1); *Prunus persica* PpCCD7 (XP_007221108.1), PpCCD8 (XP_007222386.2); *Pyrus communis* PcCCD7 (PCP005718), PcCCD8 (PCP000841); *Vitis vinifera* VvCCD7 (XP_002274198.1), VvCCD8 (XP_002281239.2).

The apple *CCD7* and *CCD8a* genes were isolated using a combination of searching an in-house EST data base, degenerate primer PCR, gene walking, and 5'/3' RACE as described previously (Ledger *et al.*, 2010). Full-length cDNAs of *MdCCD7* and *MdCCD8a* were amplified from ‘Royal Gala’ (RG) root mRNA. A second *MdCCD8* gene, hereafter referred to as *MdCCD8b*, was identified later by whole-genome sequencing. All the primers used in this study are listed in Supplementary Table S1 at *JXB* online. Amino acid sequences of MdCCD7, MdCCD8a/b, and other full-length CCD proteins were aligned using ClustalW. The phylogenetic tree shown in Figure 1 was constructed using the maximum-likelihood principle and bootstrap values of 500 replicates using Geneious Pro software version 10 (Auckland, New Zealand).

### Complementation of Arabidopsis *max3* and *max4* mutants

Full-length cDNA clones of *MdCCD7* and *MdCCD8a* were cloned into pHEX2 (Hellens *et al.*, 2005), both under the control of the *Cauliflower mosaic virus* (CaMV) 35S promoter. The overexpression constructs were transformed into *Agrobacterium tumefaciens* strain LBA4404. The *MdCCD7* overexpression construct was transformed into the Arabidopsis *max3-1* mutant, and the *MdCCD8a* overexpression construct was transformed into the Arabidopsis *max4-2* mutant using the floral dip method (Clough and Bent, 1998). Seven independent lines of *MdCCD7/max3* and eight independent lines of *MdCCD8a/max4* were generated. T_2_ seeds were selected on kanamycin in tissue culture then transferred to the glasshouse and grown under long days (16 h light:8 h dark). Control plants containing pHEX2 (an empty vector control) were germinated on kanamycin, and *max3-1* and *max4-2* seeds were germinated in tissue culture without antibiotic selection. The number of rosette branches and primary shoot length were recorded 10 weeks after planting, once flowering was complete. Between 5 and 16 biological replicates of each independent transformation line were phenotyped. Mean rosette branch number was compared by ANOVA followed by Fisher’s protected least significant difference (LSD) multiple comparison test to determine if the *MdCCD7* and *MdCCD8a* lines were able to complement the *max3-1* and *max4-2* mutant phenotypes, respectively (GenStat 17th edition, VSN International, Hemel Hempstead, UK).

### Transgenic RNAi knockdown of *MdCCD7* and *MdCCD8* in apple and phenotype measurements

To generate RNAi knockdown lines of *MdCCD7* and *MdCCD8* in apple, a 424 bp fragment from the RG cDNA sequence of *MdCCD7* and a 482 bp fragment from the RG cDNA sequence of *MdCCD8a* were both cloned as inverted repeats into the vector pTKO2 (Snowden *et al.*, 2005) to produce RNAi constructs for each gene (Supplementary Table S2). It was assumed that the *MdCCD8a* construct could knock down both *MdCCD8a* and *MdCCD8b.* These constructs were transformed into *A. tumefaciens* strain LBA4404 and then used to generate nine independent lines of *MdCCD7* RNAi and 15 of *MdCCD8* RNAi using the protocol described by Yao *et al.* (1995). Regenerated shoots were subcultured and maintained on selective media for 5 months. Eight untransformed RG plants that were regenerated in tissue culture were used as controls. In late 2008 to early 2009 (New Zealand summer), plants were transferred into potting mix and grown in a glasshouse under natural light and ambient temperature.

In the spring of 2010, all trees were cut back to 20 cm and one shoot was allowed to regenerate into a primary axis. After growth had terminated, the length and node number of the primary axis and any sylleptic shoots were recorded for each RNAi line and seven RG trees. Average internode length was determined by dividing primary axis length by node number. Some trees flowered and set fruit in 2013, and examination of the fruit suggested a possible increase in number of locules and seeds in the RNAi lines. In spring of 2014, flowers were hand pollinated to increase fruit set. In autumn of 2015, a total of 5–25 fruit from each line and from eight RG trees were cut in half, photographed, and locule and seed number recorded. Leaf blade length and width and petiole length were measured on six fully expanded leaves taken from at least three different sylleptic shoots. Graphing of data was performed in Excel^®^ 2013 (Microsoft Corporation, Redman, WA, USA).

### Quantitative real-time PCR

To verify reduced expression of *MdCCD7* and *MdCCD8* in the RNAi lines, root tissue was collected in early summer from transgenic and control plants 3 months after transfer of the plants to the glasshouse. Only 7 of the 15 *MdCCD8* lines had enough roots to remove some for RNA isolation without jeopardizing the health of the plants. The *MdCCD8* primers detected the combined expression of both *MdCCD8a* and *MdCCD8b.* RNA was also extracted from WT RG leaves, roots, open flowers, young fruits [14 days after full bloom (DAFB)], mature fruit (146 DAFB), and stem tissue to determine expression levels of *MdCCD7* and *MdCCD8* in these tissues. To collect stem tissue, a section of bark was removed from a field-grown RG tree, the inside of the bark was sampled for phloem- and cambium-enriched tissue, and the tree side was sampled for xylem-enriched tissue (Fig. 3; all values are relative to *MdCCD8* expression in roots). To measure *MdCCD7* expression in roots of grafted trees, root tissue was collected from 4–5 biological replicates of each scion–rootstock combination from trees grown in 2014–2015. All tissue was snap-frozen in liquid nitrogen and stored at –80 °C until RNA extraction. Total RNA was extracted using a cetyltrimethylammonium bromide (CTAB) method as modified by Ledger *et al.* (2010). The extracted total RNA was treated with DNase I and the quality and concentration of RNA were determined using either an Agilent 2100 Bioanalyser (Agilent Technologies, Palo Alto, CA, USA) or a Fragment Analyser (Advanced Analytical, Ankey, USA). Only RNAs with an RNA integrity number of ≥8.0 were made into cDNA. First-strand cDNA was synthesized from 1.0 µg of total RNA using poly(dT) primer and Primescript Reverse Transcriptase (TaKaRa, Kusatsu, Japan).

**Fig. 2.**
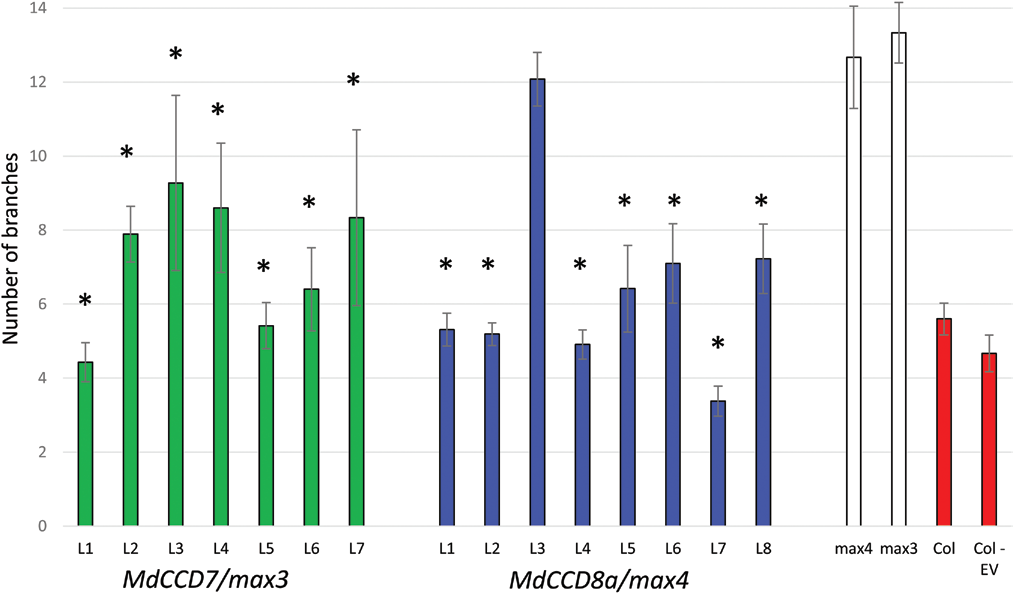
Complementation of Arabidopsis branching mutants with *MdCCD7* and *MdCCD8a*. Number of rosette branches in seven independent transgenic lines of 35S:*MdCCD7* in *max3-1* and eight lines of 35S:*MdCCD8a* in *max4-2* compared with *max3-1*, *max4-2*, wild-type ‘Columbia’ (Col) Arabidopsis, and plants containing an empty pHEX vector (Col-EV). Values are means ±SE, *n*=5–16 for each independent line. Means were compared using ANOVA, followed by Fisher’s protected LSD multiple comparison test; an asterisk above the bar indicates a significant difference from *max3-1* and *max4-2* at *P*≤0.05.

**Fig. 3.**
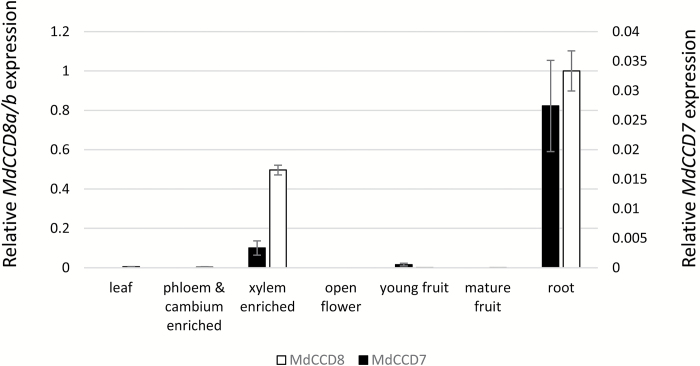
Expression of *CAROTENOID CLEAVAGE DIOXYGENASE* (*CCD*) genes in ‘Royal Gala’ apple. qRT-PCR expression of *MdCCD7* and *MdCCD8* in wild-type tissues. Values are means of three technical replicates ±SE, normalized to internal control genes and relative to *MdCCD8* expression in roots. Tissues without bars had expression levels below the threshold of detection. The scale for *MdCCD7* is on the right axis and for *MdCCD8* is on the left.

Quantitative real-time PCR (qRT-PCR) was performed with KAPA sybr fast qRT-PCR mastermix (Wilmington, MA, USA) on a Roche 480 LightCycler^®^ (Basel, Switzerland). cDNAs were diluted 1:20 and run in three technical replicates, with 1.0 µl of template in a reaction volume of 7.5 µl. PCRs were set up in a 384-well plate by a Biomek liquid handling robot (Waltham, MA, USA) to minimize pipetting errors. PCR cycles were as follows: initial denaturation at 95 °C for 5 min, followed by 45 cycles of 94 °C 10 s, 55 °C 15 s, 72 °C 10 s, and a final melt curve analysis to determine if a single product was amplified. Primers were designed by Primer 3 (http://www.bioinformatics.nl/cgi-bin/primer3plus/primer3plus.cgi) to amplify products of 100–120 bp (Supplementary Table S1). For each analysis, a no-cDNA template was included as a negative control and actin, glyceraldehyde phosphate dehydrogenase (GAPDH), and EST 32701 were used as reference genes. Primer efficiencies and relative expression for three technical replicates per sample were calculated using Roche 480 Light Cycling software version SW1.5 (Basel, Switzerland). One-way ANOVA and graphing were performed with OriginLab 8.5 (Northampton, MA, USA).

### Clonal propagation of scion and rootstock material

The *MdCCD7* RNAi line AS3354 was selected for the reciprocal grafting experiments. The AS3354 tree (hereafter referred to as *ccd7* for simplicity) and WT RG stems were clonally propagated by cutting into segments, grafting onto donor rootstocks, and inducing the *ccd7* and RG scions to root using an aerial layering technique. In late winter (August), segments of *ccd7* and WT stems were cleft grafted onto donor rootstocks and 2–3 shoots grew out per plant. In January, the lower leaves of the shoots (~20 cm) were removed, the stem base was girdled, and the wound was treated with indole-3-butyric acid (1000 mg l^-1^) to induce rooting. Wet sphagnum moss was placed over the girdle and wrapped securely within plastic. Holes were punctured in the plastic to allow aeration before aerial layers were covered with aluminium foil to prevent overheating. By autumn, roots had formed around the base of the *ccd7* and WT scions. These rooted shoots were excised from the donor rootstock while dormant, and roots were bedded into moist sawdust until winter grafting.

### Grafting experiments and data analysis

Scions and rootstocks of uniform diameter (8–12 mm) were grafted in August 2013, 2014, and 2016 using standard bench grafting techniques (Seleznyova *et al.*, 2003). Root restriction is known to limit sylleptic branching (van Hooijdonk, 2009), therefore trees were grown in 50 litre black polythene planter bags spaced at least 1 m apart, watered by an automated system, under natural light in a glasshouse. The amount of *ccd7* material limited the number of grafted trees that could be generated in any year. Any *ccd7* material not used in the first experiment was propagated to generate more scions and rootstocks for the second and third experiments. Throughout this paper, the scion genotype is given before that of the rootstock (scion/rootstock). In the first experiment (2013–2014), we generated three *ccd7/ccd7*, four *ccd7/*WT, three WT/*ccd7*, and four WT/WT control trees. In the second experiment (2014–2015), we generated three *ccd7/ccd7*, five *ccd7/*WT, four WT/*ccd7*, and five WT/WT control trees. For the first two experiments, phenotyping began once the scion bud opened and the primary shoot began to extend. Primary shoot length and node number, sylleptic shoot node number, and the nodal position on the primary axis were recorded every 4 weeks in 2013–2014 and every 2 d in 2014–2015 until shoot growth terminated. In the third experiment (2016–2017), five trees each of WT/WT and *ccd7/*WT were generated. These were measured for final primary shoot length, node number, and position of sylleptic shoots along the primary axis. Graphing and one-way ANOVA were performed in OriginLab 8.5. Lines on the graphs shown in Fig. 7 and Supplementary figures are connected with B-spline. Maximum primary shoot growth rates were estimated by fitting a Boltzmann growth function to smoothed growth data for each treatment, and the maximum growth rates were calculated from the fitted functions. Unlike shoot length, node appearance is a discrete integer, which complicates calculation of the growth rate in terms of nodes d^–1^.

## Results

### Identification of *MdCCD7* and *MdCCD8a/b*

A partial clone of a *CCD8* gene was identified in an in-house apple EST database, but no sequences with homology to *CCD7* genes were found. The full-length apple *MdCCD7* and *MdCCD8a* genes were identified using a combination of degenerate primer PCR, genome walking, and 5'/3' RACE. The full-length cDNA clone of *MdCCD7* has an ORF of 1842 bp (MF034498) and corresponds to two truncated gene models on linkage group (LG) 2 (MDP0000197409 and MDP0000139334). We identified one *MdCCD8* gene (identical to XP_008378214.1), and whole-genome sequencing revealed a second gene (XP_008352014.1), consistent with *Malus* being an allopolyploid (Evans and Campbell, 2002; Xiang *et al.*, 2017). The two *MdCCD8* genes are 94.5% homologous to one another at the amino acid level, suggesting that they are homeologous genes rather than different alleles of the same gene, which tend to be 98–99% homologous (Foster *et al.*, 2007; Chagné *et al.*, 2012). Both *MdCCD8* cDNAs have an ORF of 1692 bp. *MdCCD8a* (XP_008378214.1) corresponds to MDP0000227870 on LG8, and *MdCCD8b* (XP_008352014.1) corresponds to MDP0000219296 on LG15. The chromosomal regions containing MDP0000227870 and MDP0000219296 have been duplicated, providing further support that these are homeologous genes (Velasco *et al.*, 2010).

The predicted MdCCD7 and MdCCD8a/b amino acid sequences were used in a phylogenetic analysis of the CCD family (Fig. 1). This included full-length CCD7 and CCD8 protein sequences from other plants and AtCCD1 and AtCCD4 as outgroups. The *MdCCD7* gene is in the clade including the Arabidopsis, pea, petunia, and rice *CCD7* genes identified by the branching mutants *max3*, *rms5*, *dad3*, and *d17*/*htd1*, and the predicted orthologues from other species. The *MdCCD8* gene is in the clade that includes Arabidopsis, pea, petunia, and rice *CCD8* genes identified by the *max4*, *rms1*, *dad1*, and *d10* branching mutants and the predicted orthologues from other species. Both MdCCD7 and MdCCD8a/b cluster closely with the respective CCD proteins from the *Rosaceae* family.

### *MdCCD7* and *MdCCD8* complement the Arabidopsis branching mutants *max3* and *max4*, respectively

To determine if the CCD protein function is conserved between apple and Arabidopsis, we transformed *MdCCD7* into the Arabidopsis *max3* mutant and *MdCCD8a* into *max4*. The apple genes were introduced into the Arabidopsis mutants under the control of the CaMV 35S promoter. Mean rosette branch number was compared by ANOVA followed by Fisher’s protected LSD multiple comparison test. All seven of the *MdCCD7* lines and seven of the eight *MdCCD8a* independent lines had significantly fewer rosette branches than either *max3* or *max4* (Fig. 2). The apple genes were able to complement the Arabidopsis branching mutants functionally, indicating conserved CCD7 and CCD8 function.

### *MdCCD7* and *MdCCD8* transcripts are most abundant in roots

We measured *MdCCD7* and *MdCCD8* transcript abundance in apple using qRT-PCR. RNA was isolated from a range of WT apple tissues and developmental stages. The highest level of both *MdCCD7* and *MdCCD8* expression was in roots (Fig. 3). Expression of both genes was also detected in xylem-enriched stem tissue, but was negligible in leaf, flowers, fruit, and phloem/cambium-enriched stem tissue. The abundance of the *MdCCD8* transcript was >13- and 20-fold that of *MdCCD7* in roots and xylem-enriched stem tissue, respectively.

### Knockdown of *MdCCD7* expression in apple results in increased sylleptic branching

**Fig. 4.**
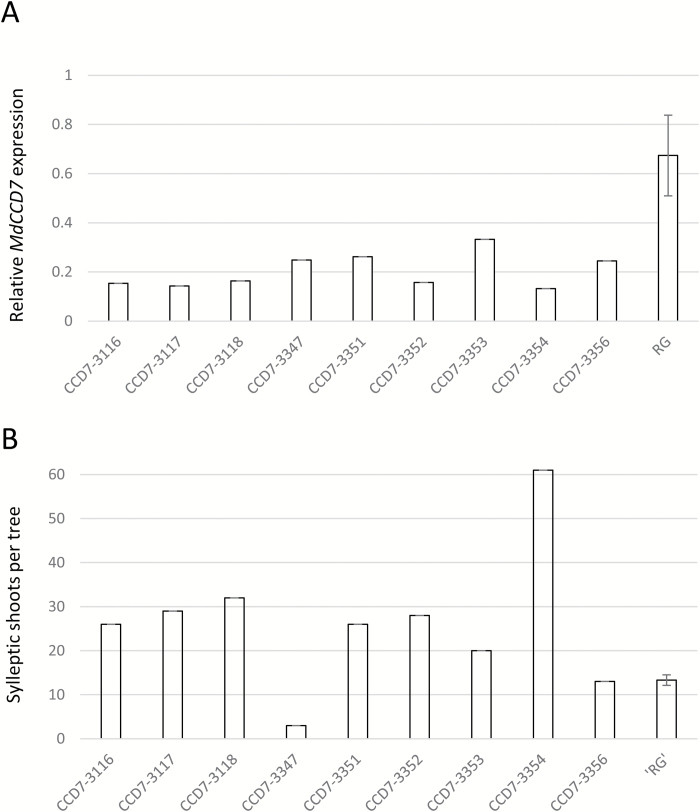
Expression of *MdCCD7* and sylleptic shoot number of *MdCCD7* RNAi ‘Royal Gala’ (RG) apple lines. qRT-PCR expression of (A) *MdCCD7* in the roots of nine individual *MdCCD7* RNAi lines and (B) total number of sylleptic shoots per tree in nine individual *MdCCD7* RNAi lines relative to RG controls. For the controls, values are the means of eight biological replicates of RG ±SE.

Transgenic RNAi lines of *MdCCD7* and *MdCCD8* were generated in RG apple to determine the effect of reduced CCD expression on sylleptic branching. Nine independent lines of RNAi *MdCCD7*, 15 of RNAi *MdCCD8*, and eight control RG trees were generated. Because of the time required to obtain progeny or even propagate apple trees clonally, each line consisted of the original transformed tree. The transcript abundance of *MdCCD7* and *MdCCD8* in the roots of transgenic RNAi lines was determined 3 months after transfer to the glasshouse. Transcript abundance of *MdCCD7* in individual RNAi lines was 2- to 5-fold lower than in RG controls and very consistent between RNAi lines (Fig. 4A). The transcript abundance of *MdCCD8* was reduced in only three of the eight RNAi lines tested, suggesting that the RNAi construct was not very effective in reducing *MdCCD8* expression (Supplementary Fig. S1A). The hairpin sequence was identical to that of *MdCCD8a*, but slightly more divergent to the *MdCCD8b* gene model (Supplementary Table S2).

In the spring of 2010, trees were cut back to 20 cm and a single primary shoot was allowed to develop from an axillary bud. Final shoot length and node number were recorded for primary and sylleptic shoots once growth had terminated. With the exception of two lines, all of the *MdCCD7* RNAi lines had more sylleptic shoots than the controls (Fig. 4B). In contrast, only 4 of the 15 *MdCCD8* RNAi lines had more sylleptic shoots than the controls (Supplementary Fig. S1B). Both *MdCCD7* and *MdCCD8* RNAi tended to have slightly shorter primary axes and had fewer nodes than controls (Supplementary Fig. S2). The *MdCCD8* RNAi lines did not show greatly reduced *MdCCD8* expression or increased branching, and therefore we focused on more detailed analysis of sylleptic branching in the *MdCCD7* RNAi lines.

### Fruit and leaf phenotypes of RNAi lines

The petunia *dad1* and *dad3* mutants (mutations in *CCD7* and *CCD8*, respectively) produce about half the seed yield per capsule as the WT (Simons *et al.*, 2007). Some of the RNAi trees first fruited in 2013, and a preliminary investigation indicated that several lines had fruit with an abnormal number of locules and seeds (Supplementary Fig. S3A). A more detailed analysis of fruit in 2015 indicated that locule numbers in most of the RNAi lines were similar to those of RG, but the number of seeds per fruit were reduced in about half the *MdCCD7* and *MdCCD8* lines (Supplementary Fig. S3B). Previous studies have found that SL-deficient mutants have altered leaf morphology (Beveridge *et al.*, 1997; Scaffidi *et al.*, 2013; Lauressergues *et al.*, 2015). There was much more variability in leaf dimensions in about half the RNAi lines relative to that in RG, but no consistent trend was observed (Supplementary Fig. S4).

### Grafting experiments demonstrate that *MdCCD7* expression in the scion, but not the rootstock, supresses branching

Grafting experiments were performed to determine whether reduced *MdCCD7* expression in roots or shoots altered sylleptic shoot outgrowth. Sylleptic branching is strongly influenced by tree spacing, so these experiments required a large containment glasshouse. Initiating roots on woody material is technically difficult, which also limited the number of trees that could be grown each year. *MdCCD7* RNAi line AS3354 had the lowest transcript abundance of *MdCCD7* and the highest number of sylleptic shoots. This line, hereafter referred to as *ccd7*, was selected for clonal propagation and subsequent grafting experiments. Four combinations of scion and rootstock were created between *ccd7* and WT RG, including *ccd7* and WT homografts (*ccd7/ccd7* and WT/WT, respectively). During the 2013–2014 season, primary and sylleptic shoot growth was measured monthly until the cessation of growth.

At the beginning of the season when the lower nodes of the primary shoot were extending, the *ccd7/ccd7* trees had the highest cumulative sum of sylleptic shoots per tree (Supplementary Fig. S5A). There was a slight effect of WT rootstocks at the base of *ccd7* scions, but, as the uppermost nodes developed towards the end of the season, the *ccd7*/WT (scion/rootstock) trees developed the greatest number of sylleptic shoots per tree; that is, WT rootstocks failed to supress branching in *ccd7* scions. By the end of the season, WT/*ccd7* trees developed the same number of sylleptic shoots as did the WT/WT trees, both of which were significantly fewer than on the *ccd7/*WT and *ccd7/ccd7* trees (Supplementary Fig. S5A). Grouping the data by scion or rootstock genotype, it was clear that trees with *ccd7* scions had a significantly higher number of sylleptic shoots than those with WT scions (Supplementary Fig. S5B). Moreover, rootstock genotype had no significant effect on final sylleptic shoot number (Supplementary Fig. S5C). Interestingly, the scion genotype effect became greater towards the top of the primary axis. Sylleptic shoots on *ccd7* scions had twice as many nodes as sylleptic shoots on WT scions (Supplementary Fig. S6A).

**Fig. 5.**
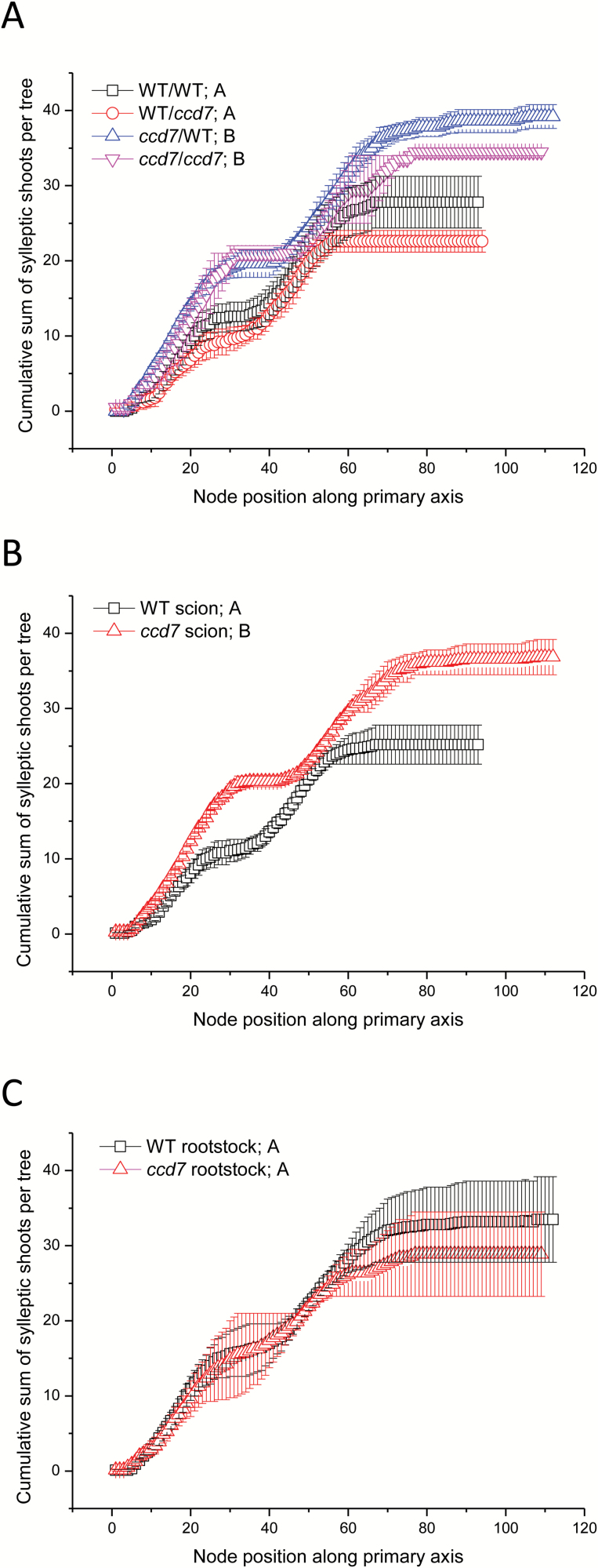
The cumulative sum of sylleptic shoots per ‘Royal Gala’ apple tree for each graft combination in 2014–2015. For each tree, the presence or absence of a sylleptic shoot was recorded at each node along the primary axis from the base to the tip. The cumulative sum at each node for (A) all four graft combinations (scion/rootstock), (B) trees grouped by scion genotype, and (C) rootstock genotype. Symbols are means of 3–5 biological replicates ±SE. Means were compared by one-way ANOVA; different letters after each symbol in the key represent a significant difference at *P*≤0.05. WT, wild-type ‘Royal Gala’.

The experiment was repeated in 2014 with a new set of grafted trees to confirm the consistency of our results. Similar trends were observed in the second experiment. WT rootstocks were unable to suppress sylleptic branching in *ccd7* scions, even at the lowest nodes (Fig. 5A). Regardless of rootstock genotype, the *ccd7* scions developed significantly more sylleptic shoots, especially at the upper nodes of the primary axis (Fig. 5A). Once again, the genotype of the scion, not the rootstock, determined the cumulative sum of sylleptic shoots (Fig. 5B, C). All trees showed two distinct flushes of sylleptic shoot development; however, the first flush was much more pronounced in the *ccd7* scions (Fig. 5B). Sylleptic branches had twice as many nodes and occurred higher on the primary axes of *ccd7* scions relative to WT scions (Supplementary Fig. S6B).

**Fig. 6.**
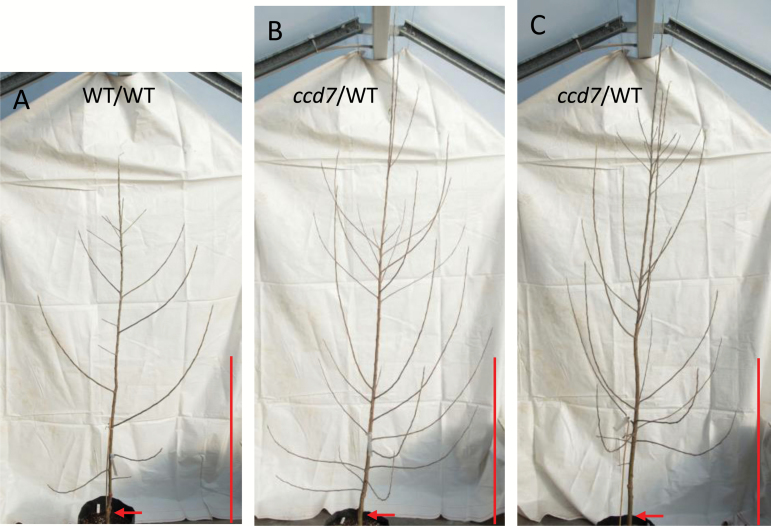
WT/WT and *ccd7*/WT ‘Royal Gala’ apple trees grown in 2016–2017. Photographs of a representative (A) WT/WT tree and (B, C) *ccd7*/WT trees (scion/rootstock); the red bar represents 1 m and red arrows show graft junctions. WT, wild-type ‘Royal Gala’.

Only WT/WT and *ccd7/*WT trees were generated in 2016. Sylleptic shoot position and final primary axis length were recorded at the end of the growing season. Consistent with data from the previous 2 years, *ccd7*/WT trees developed significantly more sylleptic shoots than WT/WT trees, especially at the upper nodes of the primary axis (Fig. 6; and Supplementary Fig. S7).

### Reduced expression of *MdCCD7* in the scion increases primary axis growth rate

**Fig. 7.**
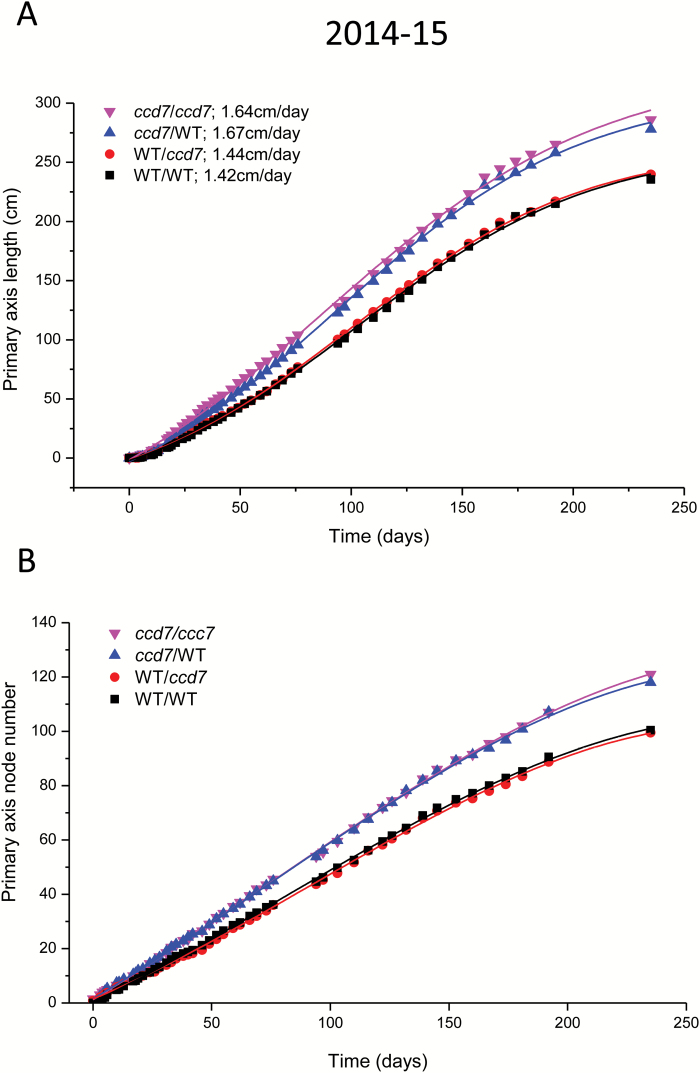
Growth of the primary axis for each ‘Royal Gala’ apple graft combination (scion/rootstock) in 2014–2015. Growth was measured in terms of increase in (A) primary axis length and (B) node number over the growing season. The maximum growth rate (cm d^–1^) was estimated by fitting a Boltzmann function to the smoothed growth data and is listed after each symbol in the key. WT, wild-type ‘Royal Gala’.

In all three grafting experiments, we found that *ccd7* scions had a significantly larger final node number and primary shoot length than wild-type scions (Supplementary Fig. S8). To determine if this resulted from a longer period of growth or a higher growth rate, we recorded the node number and length of the primary shoot throughout the growing season for the first two grafting experiments. Primary shoot growth rate, in terms of both increase in length and node initiation, showed a similar trend in both years. The *ccd7/ccd7* and *ccd7/*WT trees had a higher primary shoot growth rate than WT/*ccd7* and WT/WT trees (Fig. 7; Supplementary Fig. S9). The maximum growth rate of *ccd7* scions was ~15% greater than that of the WT scions (Fig. 7). All trees terminated growth at about the same time. Average internode length was similar between all graft combinations in all three experiments (data not shown).

### No graft-transmissible knockdown of *MdCCD7* in wild-type roots

Our findings differ from many previous reports where *CCD* expression in the roots is able to restore wild-type branching in *CCD* mutant scions. One possible explanation for our data was that the hairpin used in the *MdCCD7* RNAi lines moved from the scion and knocked down expression of *MdCCD7* in wild-type roots. To test this, we measured *MdCCD7* transcript abundance in roots from the four graft combinations. *MdCCD7* expression was 5- to7-fold higher in WT roots than in *ccd7* roots regardless of scion genotype (Supplementary Fig. S10). This result indicates that the failure of WT scions to repress branching in *ccd7* scions is not caused by a graft-transmissible knockdown in *MdCCD7* expression in WT roots.

## Discussion

In this study, we identified the apple orthologues of *CCD7* and *CCD8* and demonstrated that they were able to complement the Arabidopsis branching mutants *max3* and *max4*, respectively. This indicates that CCD7 and CCD8 function is conserved between the woody perennial apple and the annual Arabidopsis. Reduced expression of *MdCCD7* in apple by RNAi resulted in an increased number of sylleptic shoots in the majority of lines. In contrast, only 4 of the 15 *MdCCD8* RNAi lines showed slightly increased sylleptic shoot number. The hairpin constructs were designed before whole-genome sequencing identified two copies of *CCD8* in apple. The *MdCCD8* hairpin construct included a region that was identical to *MdCCD8a*, but slightly more divergent from *MdCCD8b*; therefore, it is possible that only *MdCCD8a* was effectively silenced. Loss of either CCD7 or CCD8 has resulted in blocked SL biosynthesis and highly branched and dwarfed phenotypes in a range of plants (Beveridge *et al.*, 1997; Morris *et al.*, 2001; Turnbull *et al.*, 2002; Booker *et al.*, 2004; Ishikawa *et al.*, 2005; Snowden *et al.*, 2005; Arite *et al.*, 2007; Drummond *et al.*, 2009).

An important caveat for this work is that we were unable to generate complete knockouts of *MdCCD7* and *MdCCD8*, so we selected the RNAi line with the lowest *MdCCD7* expression for grafting experiments and detailed architectural measurements. This line showed consistent results in three independent grafting experiments, and showed an increase in sylleptic branching with a concomitant reduction in *MdCCD7* transcripts. However, the increase in nodes produced on the primary axis of *ccd7* scions (Supplementary Fig. S8) contrasted with the result obtained in the initial phenotyping of the complete set of RNAi lines (Supplementary Fig. S2). The reason for this difference is unknown; however, it is worth noting that the trees were grown in different conditions for these analyses. In particular, the complete set of RNAi lines were grown in much smaller pots with less spacing between trees than the grafted trees (5 litres versus 50 litres). In addition, the age of the trees at phenotyping, and time since removal from tissue culture differed between the experiments. Future studies to examine the role of SLs in apple tree growth would benefit from the use of a technique such as genome editing to knock out *MdCCD7* and *MdCCD8a/b* expression completely and using multiple lines in grafting experiments.

### *MdCCD7* genotype of the scion, not rootstock, determines the extent of branching in apple

Grafting experiments with *CCD* mutants or RNAi lines have consistently shown that WT roots are able to restore normal branching in scions that lack either *CCD7* or *CCD8* (Beveridge *et al.*, 1997; Morris *et al.*, 2001; Turnbull *et al.*, 2002; Booker *et al.*, 2004; Simons *et al.*, 2007; Muhr *et al.*, 2016). In contrast, our results demonstrated that WT roots were unable to suppress branching in *ccd7/*WT compared with the amounts of branching in WT/WT. It is worth noting that the difference in cumulative sylleptic shoot number between *ccd7* and WT scions became greater at the upper nodes of the primary axis. Researchers studying *CCD* genes in poplar have suggested that local SL biosynthesis in the shoot may be more important for bud inhibition in trees because they are much larger than annual plants (Czarnecki *et al.*, 2014; Muhr *et al.*, 2016). Indeed, WT scions grafted to *CCD7*- or *CCD8*-deficient roots all show a WT branching pattern in Arabidopsis, pea, petunia, and poplar, supporting the idea that scion expression of *CCD7* or *CCD8* is sufficient, but not necessary to suppress branching. Our results suggest that *MdCCD7* expression in the scion is necessary to inhibit sylleptic branching in apple.

To our knowledge, this is the first study to present a dynamic picture of sylleptic shoot outgrowth. Final branch number does not give any information about where on the primary axis branching occurs. By following the cumulative sylleptic shoot number, we were able to identify that the increase in sylleptic branching became more pronounced at the upper nodes of *ccd7* scions. We suggest that localized SL biosynthesis in the stem becomes more important at nodes further from the root system. Previous studies have shown that the propensity for sylleptic branching in both apple and pear is determined by the scion genotype (Costes and Guédon, 2002; Seleznyova *et al.*, 2012). Perhaps this reflects the input of SL signalling pathways.

### Extent of sylleptic branching may reflect differential sensitivity to SLs

Sylleptic branching is a highly plastic trait that is controlled by genetic, environmental, and developmental factors (Hallé *et al.*, 1978; Crabbé, 1987; Génard *et al.*, 1994; Wu and Hinckley, 2001; Cline and Dong-Il, 2002; Marron *et al.*, 2006;). The SL pathway is thought to integrate multiple environmental and internal signals such as light quality, nutrient availability, and input from other hormone signalling pathways to modulate growth (Domagalska and Leyser, 2011; Djennane *et al*, 2014; Janssen *et al*, 2014; Drummond *et al*, 2015; Rameau *et al.*, 2015). A study comparing poplar clones with high and low sylleptic branching found that the former had an increased sensitivity to auxin and a decreased sensitivity to cytokinin, whereas the latter showed the opposite (Cline and Dong-Il, 2002). Even within one species, there appears to be a range of sensitivity to hormones that regulate branching. RNAi lines of *MAX* genes in poplar have been shown to increase sylleptic branching greatly (Muhr *et al.*, 2016) or have no effect at all (Brunner *et al.*, 2011).

The function of CCD proteins in SL biosynthesis is conserved between poplar and apple, although there were some interesting phenotypic differences between the amiMAX4 and *MdCCD7* RNAi trees. The amiMAX4 poplar trees had 5–15 times more sylleptic shoots than WT poplar (Muhr *et al.*, 2016). Even the most highly branched *MdCCD7* RNAi line had <5-fold more sylleptic shoots than WT trees. The amiMAX4 homografted trees had 8–10 times more sylleptic shoots than WT/WT trees, whereas our *ccd7/ccd7* trees had roughly twice the number of sylleptic branches as the WT/WT trees.

The differences between the CCD knockdown phenotypes in poplar versus apple could be due to the effectiveness of the knockdown of gene expression or could reflect inherent differences in branching behaviour. The amiMAX4 construct was transformed into the hybrid *Populus×canescens*, which is a low syllepsis clone. Depending on the scion genotype and growth conditions, apple trees can have several flushes of sylleptic branching and produce up to 30 sylleptic shoots in the first year of growth. It is possible that apple trees have a higher threshold of SLs required for repression of bud outgrowth.

### Reduced *MdCCD7* expression associated with higher growth rate of the primary shoot

In addition to increased branching, CCD mutants or knockdown lines generally have a shorter primary axis and shorter internode lengths (Beveridge *et al.*, 1997; Morris *et al.*, 2001; Booker *et al.*, 2004; Simons *et al.*, 2007; Guan *et al.*, 2012; Muhr *et al.*, 2016). In contrast, we found that the primary axes of *MdCCD7* RNAi scions were longer, with more internodes than WT scions. Average internode length was unaffected by reduced *MdCCD7* expression, but the rate of growth, in terms of both length and node number, was increased relative to that of the WT. The rate of node initiation or plastochron index reflects the activity of the SAM, whereas internode elongation is due to a combination of cell division and elongation proximal to the shoot apex. de Saint Germain and co-workers have demonstrated that the artificial SL GR24 was able to restore internode length in the pea *rms1* mutant by increasing cell number, not cell length (de Saint Germain *et al.*, 2013). These authors proposed that SLs normally repress cell division in axillary meristems, but promote cell division in internodes. Based on our findings, we suggest that the reduction of SLs in *ccd7* scions resulted in more active apical and axillary meristems, possibly because of increased cell division. Whether these effects are independent or causally related remains to be investigated. Unlike annual plants, perennial plants must balance growth during multiple seasons with maintaining reserves for homeostasis during winter and early spring growth. Given the different survival goals and the role of SLs in integrating signals controlling growth, perhaps it is not surprising that some effects of SLs could be different in annual and perennial plants.

## Conclusions

We have identified *CCD7* and *CCD8* genes from apple and shown that their function is conserved. RNAi lines of *MdCCD7* had reduced *MdCCD7* expression and increased sylleptic branching in apple. Reciprocal grafting experiments with one RNAi line provided evidence that *MdCCD7* expression in the scion was necessary to restore WT sylleptic branch numbers. WT roots were unable to repress sylleptic branching in scions of this RNAi line. The growth rate of the primary axis was higher in this line than in the WT. Based on our findings, we propose that localized SL synthesis in the shoot may have a predominant role in suppressing sylleptic shoot outgrowth and primary shoot growth rate in apple.

## Supplementary data

Supplementary data are available at *JXB* online.

Fig. S1. Expression of *MdCCD8* and and sylleptic shoot number of *MdCCD8* RNAi ‘Royal Gala’ (RG) apple lines.

Fig. S2. Primary axis length and node number for *MdCCD7* and *MdCCD8* RNAi ‘Royal Gala’ (RG) apple lines.

Fig. S3. Fruit characteristics of RNAi and ‘Royal Gala’ (RG) apple fruit.

Fig. S4. Dimensions of RNAi and ‘Royal Gala’ (RG) apple leaves. 

Fig. S5. The cumulative sum of sylleptic shoots per ‘Royal Gala’ apple tree for each graft combination in 2013–2014.

Fig. S6. Total node number of sylleptic shoots. 

Fig. S7. The cumulative sum of sylleptic shoots per ‘Royal Gala’ apple tree for *ccd7*/WT and WT/WT trees in 2016–2017. 

Fig. S8. Final primary axis length and node number of grafted ‘Royal Gala’ apple trees (scion/rootstock). 

Fig. S9. Growth of the primary axis for each ‘Royal Gala’ apple graft combination (scion/rootstock) in 2013–2014. 

Fig. S10. Expression of *MdCCD7* in roots of grafted ‘Royal Gala’ apple trees. 

Table S1. PCR primers used in this study.

Table S2. Sequences for RNAi constructs and homology to the targeted gene.

## Supplementary Material

Supplementary Figures and Tables
