# Supplementary material for: Expression of MdCCD7 in the scion determines the extent of sylleptic branching and the primary shoot growth rate of apple trees
Source: J Exp Bot. 2017 Nov 28;69(9):2379–90. doi: 10.1093/jxb/erx404 (PMC5913623; doi:10.1093/jxb/erx404)
Supplement: Supplementary Figures and Tables [file erx404_suppl_supplementary_figures_and_tables.pdf]

Expression of *MdCCD7* in the scion determines the extent of sylleptic branching and primary shoot growth rate of apple trees

Toshi M. Foster, Susan E. Ledger, Bart J. Janssen, Zhiwei Luo, Revel S.M. Drummond, Sumathi Tomes, Sakuntala Karunairetnam, Chethi N. Waite, Keith A. Funnell, Ben M. van Hooijdonk, Ali Saei, Alla N. Seleznyova, Kimberley C. Snowden

Supplementary File

Supplemental Fig. 1. Expression of *MdCCD8* and *and sylleptic shoot number* of *MdCCD8* RNAi 'Royal Gala' (RG) apple lines. Real-time q-RT PCR expression of (**A**) *MdCCD8* in the roots of seven individual *MdCCD8* RNAi lines and (**B**) Total number of sylleptic shoots per tree in 15 individual *MdCCD8* RNAi lines relative to RG controls. For the controls, values are the means of eight biological replicates of RG ± SE.

Supplemental Fig. 2. Primary axis length and node number for *MdCCD7* and *MdCCD8* RNAi 'Royal Gala' (RG) apple lines. Final length and node number of the primary axis were recorded at the end of the growing season in 2011. Each symbol represents an individual tree, green diamonds are *MdCCD7* RNAi, blue triangles are *MdCCD8* RNAi, and red circles are RG.

Supplemental Fig.3. Fruit characteristics of RNAi and 'Royal Gala' (RG) apple fruit. (A) Some of the fruit that formed in 2013 had an irregular number of locules and seeds relative to RG. (B) A more detailed analysis of fruit in 2015 indicated that locule numbers in most of the RNAi lines were similar to those in RG, but the number of seeds per fruit were reduced in about half the MdCCD8 lines. Symbols represent the means of 5-25 fruit from each line and vertical and horizontal bars are ± SE of seeds per fruit and locules per fruit respectively. Green diamonds are *MdCCD7* RNAi, blue triangles are *MdCCD8* RNAi, and red circles are RG.

Supplemental Fig.4. Dimensions of RNAi and 'Royal Gala' (RG) apple leaves. Leaf width and length and petiole length were measured in six leaves taken from at least three different sylleptic shoots, with leaves taken from regions where leaves were fully expanded (i.e. not from shoot tips). Symbols represent means of six leaves per line and vertical and horizontal bars are ± SE of leaf blade width and length and petiole length and blade length respectively. Green diamonds are *MdCCD7* RNAi, blue triangles are *MdCCD8* RNAi, and red circles are RG.

Supplemental Fig. 5. The cumulative sum of sylleptic shoots per 'Royal Gala' apple tree for each graft combination in 2013-14. For each tree, the presence or absence of a sylleptic shoot was recorded at each node along the primary axis from the base to the tip. The cumulative sum at each node for (A)

all four graft combinations (scion/rootstock), (B) trees grouped by scion genotype and (C) rootstock genotype. Symbols are means of three to four biological replicates ± SE. Means were compared by one-way ANOVA; different letters after each symbol in the legend represent a significant difference at $P \leq 0.05$. WT = wild-type 'Royal Gala'.

Supplemental Fig. 6 Total node number of sylleptic shoots. Node number was recorded for each sylleptic shoot and the sum was calculated for each tree in the (A) 2013-14 and (B) 2014-15 growing season. Bars are means of biological replicates (n=3-5 for each class) ± SE. Means were compared by one-way ANOVA; different letters above bars represent a significant difference at $P \leq 0.05$.

Supplemental Fig. 7. The cumulative sum of sylleptic shoots per 'Royal Gala' apple tree for ccd7/WT and WT/WT trees in 2016-17. For each tree, the presence or absence of a sylleptic shoot was recorded at each node along the primary axis from the base to the tip. The cumulative sum at each node was calculated from final data. Symbols are means of five biological replicates ± SE. Means were compared by one-way ANOVA; different letters after each symbol in the legend represent a significant difference at $P \leq 0.05$. (black squares, WT/WT, red circles, *ccd7/ccd7,*). WT = wild-type 'Royal Gala'.

Supplemental Fig. 8. Final primary axis length and node number of grafted 'Royal Gala' apple trees (scion/rootstock). Average length and node number of each group of trees in (A) 2013-14, (B) 2014-15, and (C) 2016-17. Bars are means of biological replicates (*n*=3-5 for each class) ± SE. Means were compared by one-way ANOVA; different letters above bars represent a significant difference at $P \leq 0.05$. WT = wild-type 'Royal Gala'.

Supplemental Fig. 9. Growth of the primary axis for each 'Royal Gala' apple graft combination (scion/rootstock) in 2013-14. Growth was measured in terms of increase in (A) primary axis length and (B) node number over the growing season. The maximum growth rate (cm/day) was estimated by fitting a Boltzmann function to the smoothed growth data and is listed in the legend. Unlike shoot length, node appearance is a discrete integer, which complicates calculation of the growth rate in terms of nodes/day. WT = wild-type 'Royal Gala'.

Supplemental Fig. 10. Expression of *MdCCD7* in roots of grafted 'Royal Gala' apple trees. Real-time q-RT PCR expression of *MdCCD7* in roots from all four graft combinations (scion/rootstock). Values are the means of three to four to five biological replicates of each graft combination ± SE, normalized to internal control genes. WT = wild-type 'Royal Gala'.

Supplementary Table 1. PCR primers used in this study.

Supplementary Table 2. Sequences for RNAi constructs and homology to the targeted gene. The size of each sequence (from Royal Gala) and percentage of homology to each apple gene model (genome assembled from Golden Delicious, see https://www.rosaceae.org/) are given.

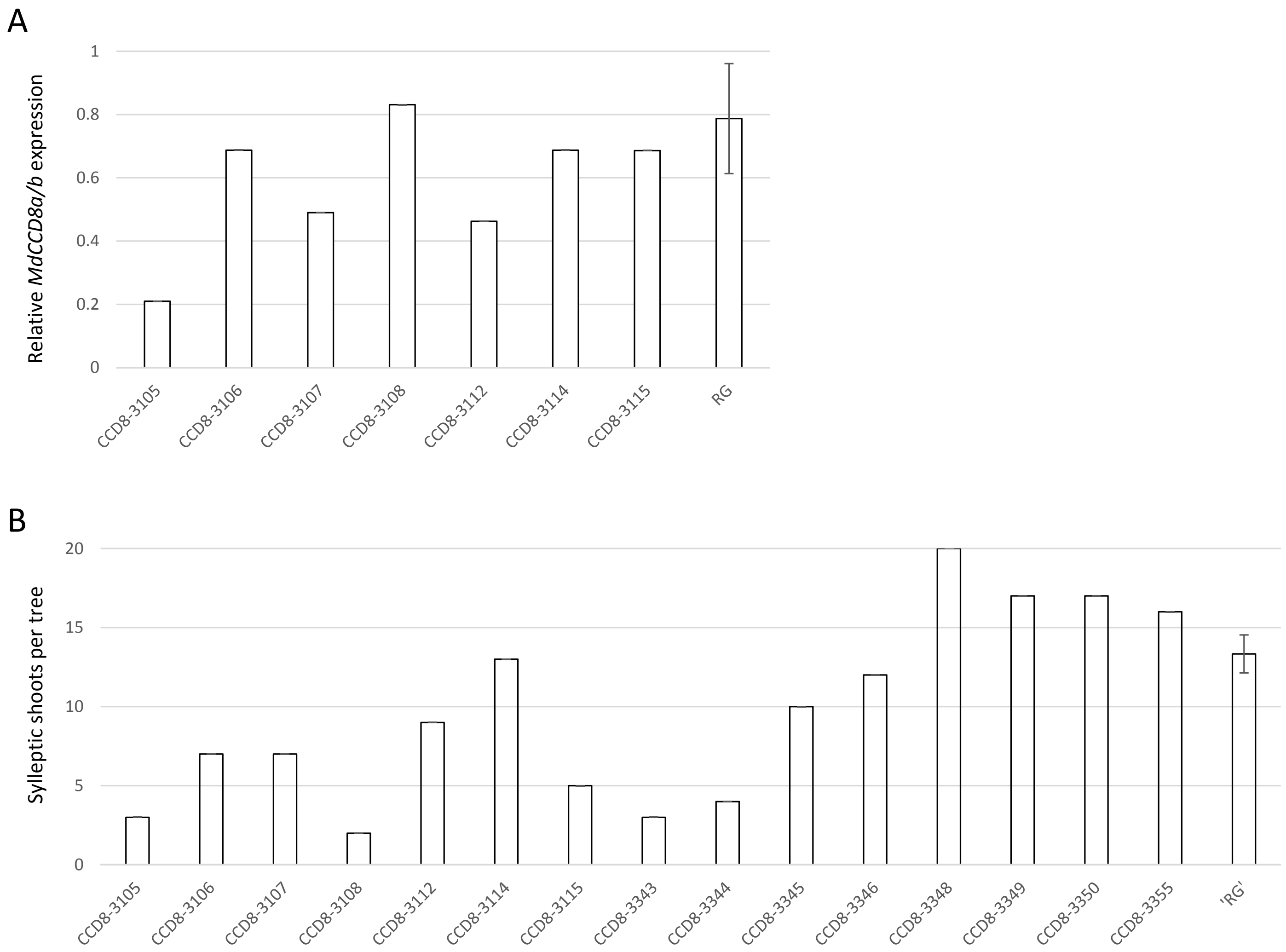

Supplemental Fig. 1. Expression of MdCCD8 and and sylleptic shoot number of MdCCD8 RNAi ‘Royal Gala’ (RG) apple lines.

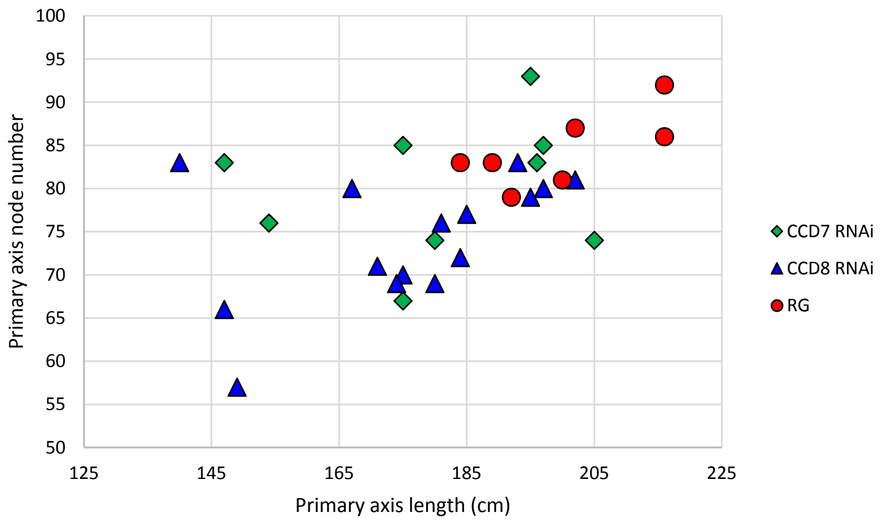

Supplemental Fig. 2. Primary axis length and node number for MdCCD7 and MdCCD8 RNAi ‘Royal Gala’ (RG) apple lines.

A

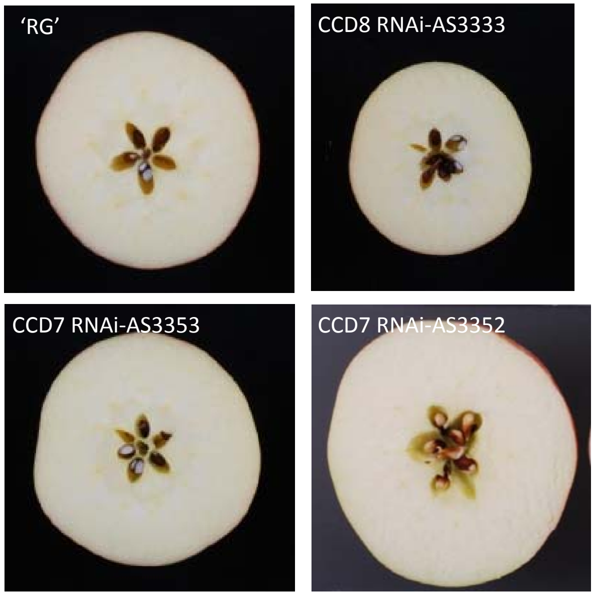

B

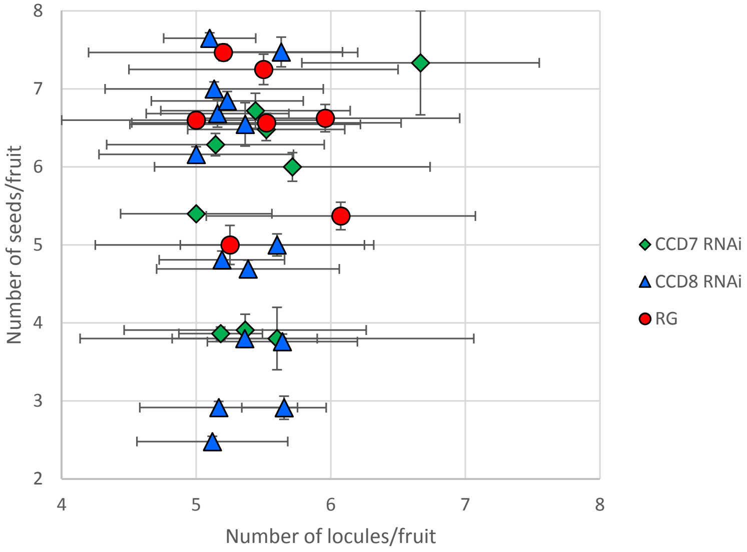

Supplemental Fig.3. Fruit characteristics of RNAi and ‘Royal Gala’ (RG) apple fruit.

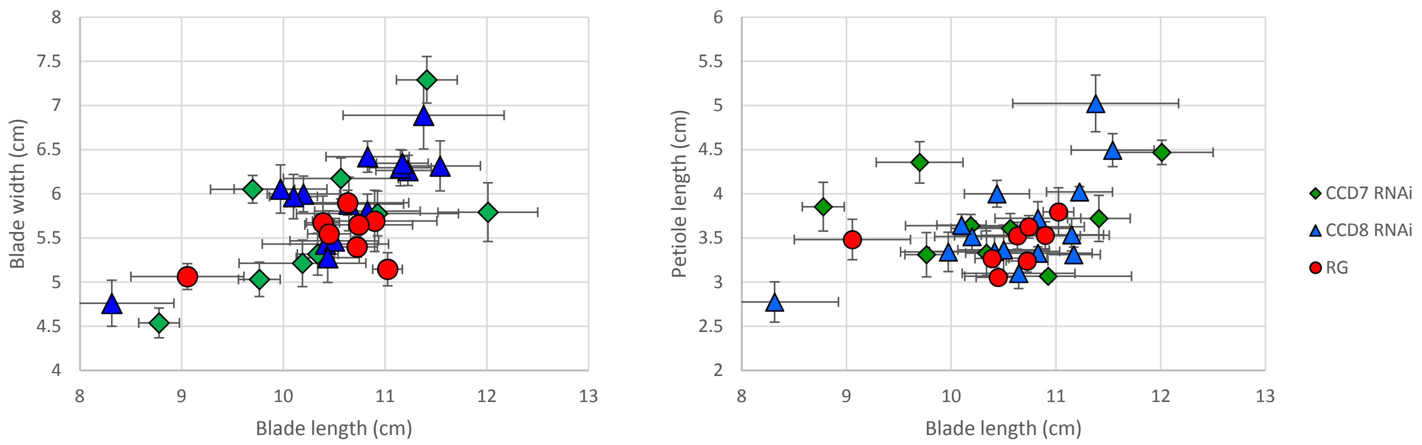

Supplemental Fig.4. Dimensions of RNAi and 'Royal Gala' (RG) apple leaves.

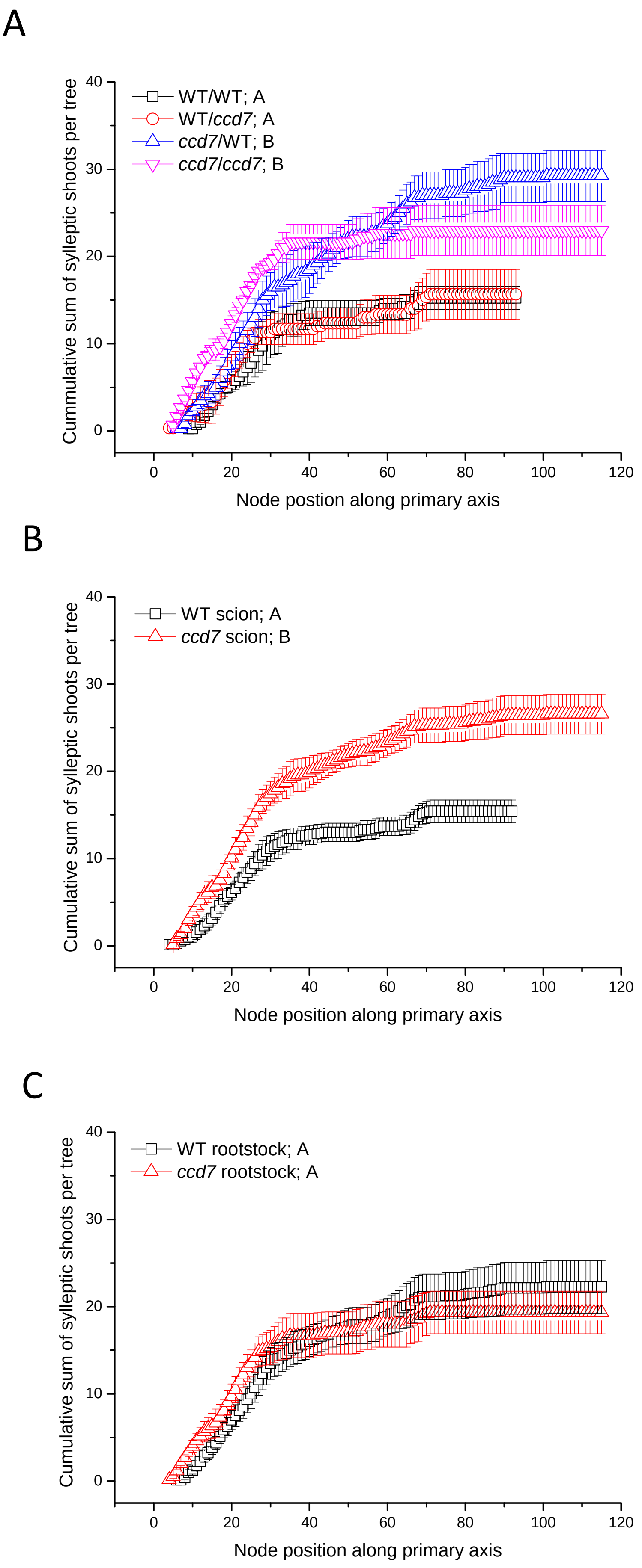

Supplemental Fig. 5. The cumulative sum of sylleptic shoots per 'Royal Gala' apple tree for each graft combination in 2013-14.

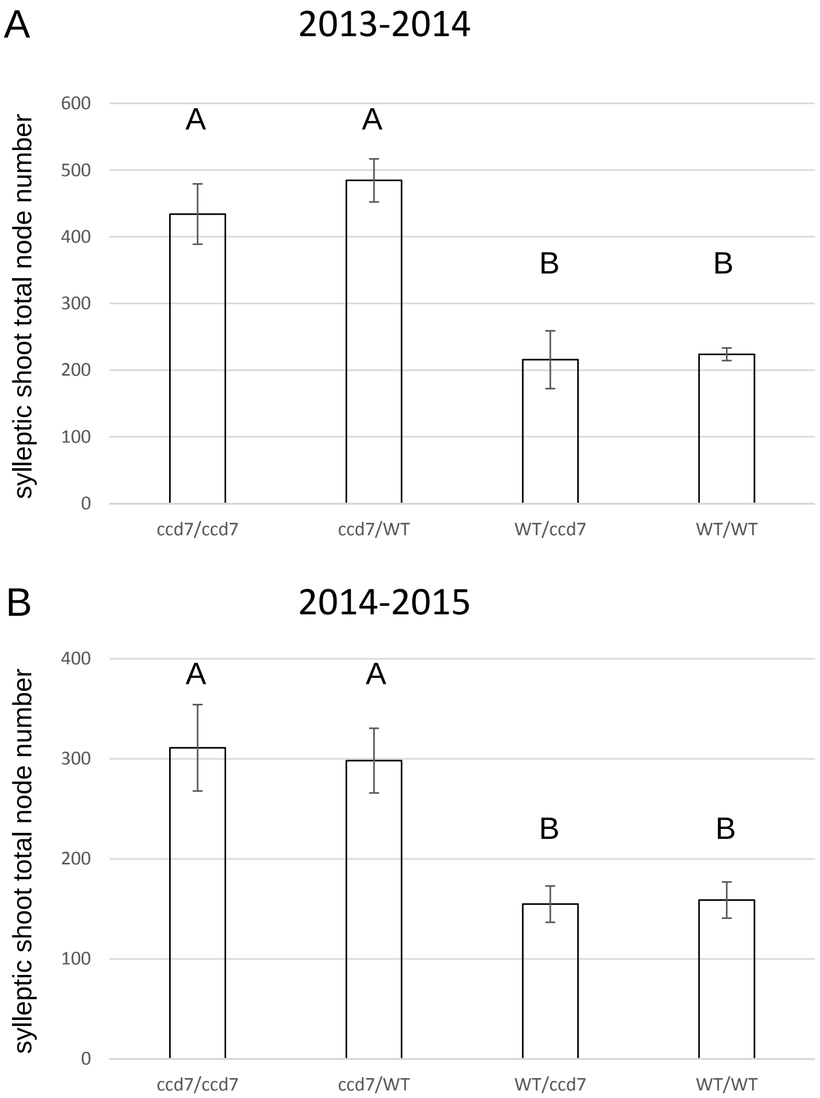

Supplemental Fig. 6 Total node number of sylleptic shoots.

2016-17

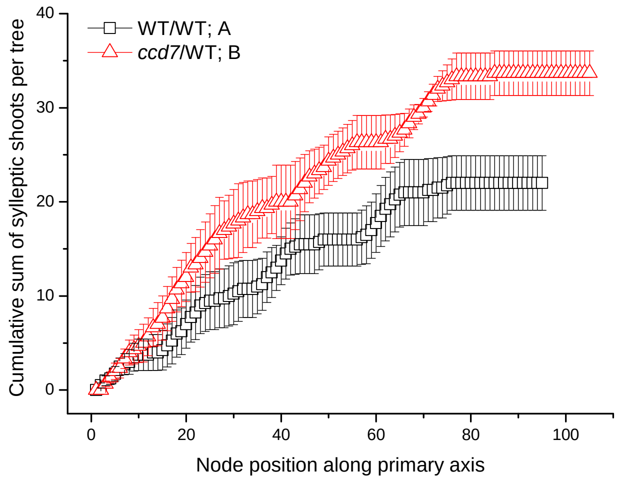

Supplemental Fig. 7. The cumulative sum of sylleptic shoots per ‘Royal Gala’ apple tree for ccd7/WT and WT/WT trees in 2016-17.

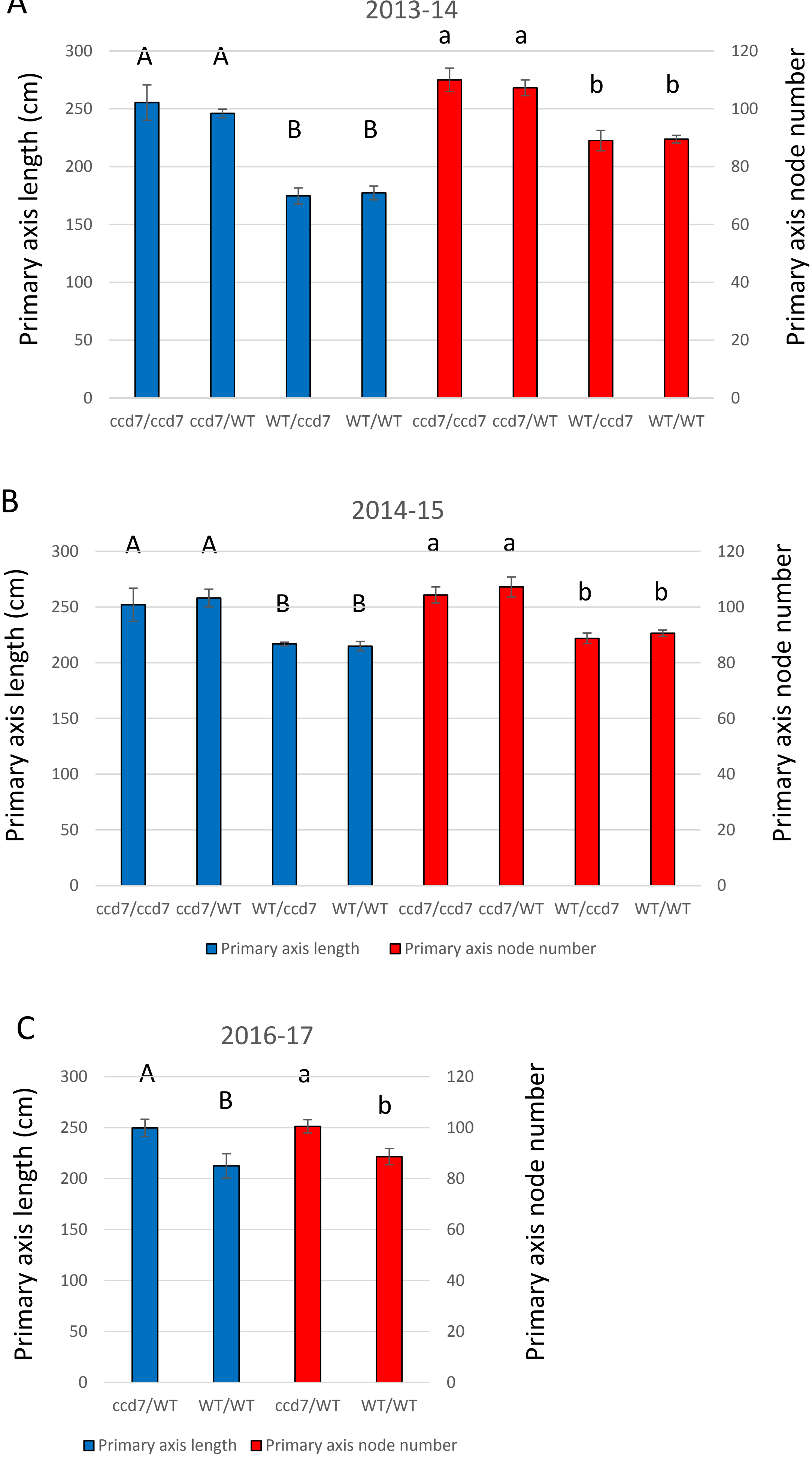

Supplemental Fig. 8. Final primary axis length and node number of grafted 'Royal Gala' apple trees (scion/rootstock).

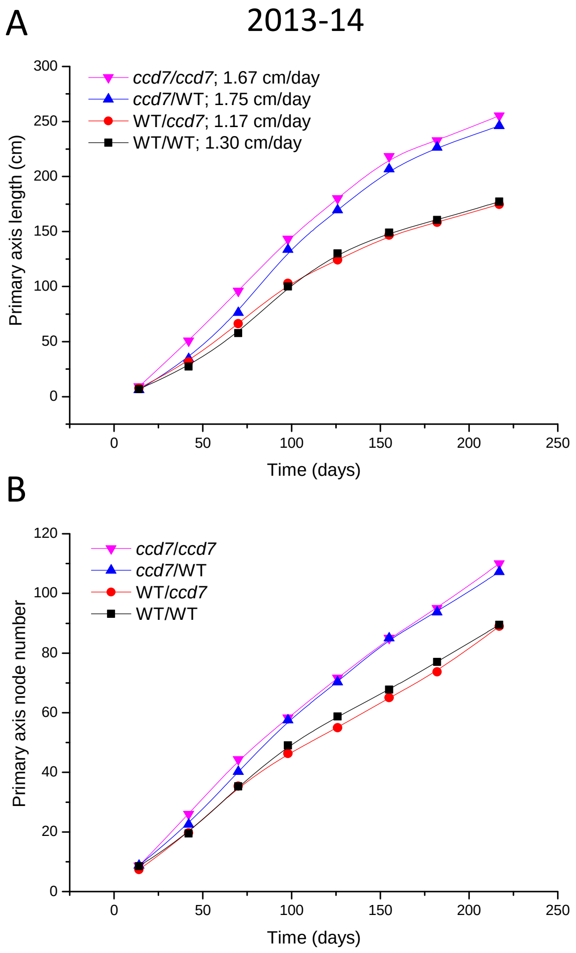

Supplemental Fig. 9. Growth of the primary axis for each 'Royal Gala' apple graft combination (scion/rootstock) in 2013-14.

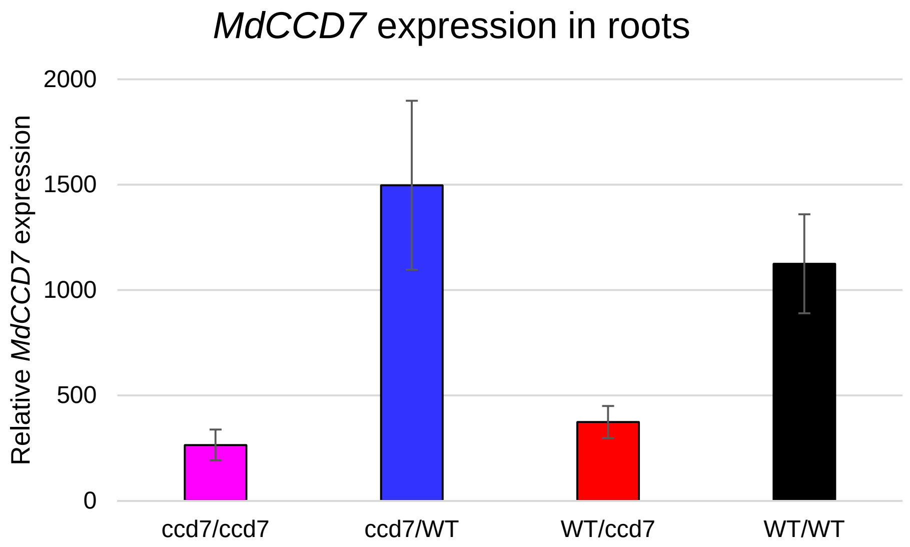

Supplemental Fig. 10. Expression of MdCCD7 in roots of grafted ‘Royal Gala’ apple trees.

Gene name	Primer sequence (5' to 3')
MdCCD7	CGGCTACCTTAGGGCTTTTC
	CTGTCAGTCGTAGGGTCGTG
MdCCD8	CAAACGTAGCAAGTCCACCA
	TCTCCTTCCCATCTCTCCTG
Actin	TGACCGAATGAGCAAGGAAATTACT
	GATGTGGATTGCCAAAGCTGAGTA
GAPDH	CTGGAATTGCATTGAACGAT
	GCTACGTGCACGATCAAGTC
EST32701	TGGAGCTCTTGTCACTGGTT
	GTGAGGTCGCAAGTTCGAG
EST262375	AACAGAAGCTGCCGGAAC
	TTATAGCGGTCACAGCATCC

Supplementary Table 1. PCR primers used in this study.

**Gene targeted**	**IASMA Gene model**	**% Identify to IASMA gene model**	**Size (bp)**	**Sequence cloned into pTKO2 (5' to 3')**
MdCCD7	MDP0000197409	100	424	ACCTGTCAGTCGTAGGGTCGTGCTC<br>TTCAACTTGAGCCTCAGTTTTCACGT<br>ACTTAGCCATGAACTTGACTTCTCCG<br>CCAAAGCCATCAAACTGAAAAGCCC<br>TAAGGTATCCGTGACCGTCTAATGG<br>GTGCACCGTCGAGCCATGGTCGTCG<br>CTGAAGAGCCCCGGCCCTGTCAAGT<br>AGTATGTGCCAGAGGGAAAGTCCGG<br>TGGAACCGAACCCTCCACGACTCTG<br>AGCGTGATGGGTTCAGTTTGTTCGG<br>AGCGTTGGGACACAAAGAGAAATTG<br>GTAGTCCCAAAATGCAGCCACTGAG<br>TCATCAACAACATCTACGGTTGATG<br>AAATAATAGGAGCATGATCGGTTTC<br>ACCGGGCGTGGAGATGGATATCGCA<br>CGCGGTGGCTTGGTGGGTGGTGGTG<br>ACAGAGATCGGTGAACCGGAGG
MdCCD7	MDP0000139334	100		
MdCCD8	MDP0000227870 (*MdCCD8a*)	98.8	482	GATCACCTTCCTCTCATTAGTACCCG<br>GCTTCATCCTGACCGCTAAATACCC<br>CGGGTTGATTAAATCCGGCAACAAA<br>GTCAAAAACTCGGTATCGGTCACAA<br>TAGGATGTGCCGAGTGTATCAAACC<br>ACCCAATGTGTCACTGTACTCAAAT<br>TTTCCCAAAGTGTCCAACGTTGTCG<br>GGTCAATCACTATTGATCCCTTCTGG<br>GTTTCTGTGGGGCACACAACCCGAC<br>CATCACCTAGCTTAACCACTCCGGT<br>GTTGGCGTTGTCAGTCAACGATGCA<br>CCGGAAAATAAGTTGGCTAGTTCTC<br>CAATATAAGATAGGAAATTGGCTGG<br>CTTAGGGACCTCAGAGAATTCACGG<br>AAACATAATTTTTGGTTCTTCATGGC<br>GGCTGTGTAGGCCTCGGATTCGATC<br>TGGCGGTGGCCAGCAGTGAGGCGGC<br>CGTCGTGGAAGTGGAGTTTGGCGAG<br>CATGGCGTAGCCGTCGAAGAGATGC<br>CGGA
MdCCD8	MDP0000219296 (*MdCCD8b*)	91.5		

Supplementary Table 2. Sequences for RNAi constructs and homology to the targeted gene. The size of each sequence and percentage of homology to each IASMA gene model are given.
